# “Downy hair sign”—A clue to autoimmune disease in curly hair

**DOI:** 10.1016/j.jdcr.2024.06.018

**Published:** 2024-07-06

**Authors:** Li-Chi Chen, Crystal Aguh

**Affiliations:** a Department of Dermatology, Lahey Hospital & Medical Center, Burlington, Massachusetts; b Department of Dermatology, Johns Hopkins School of Medicine, Baltimore, Maryland

**Keywords:** alopecia areata, autoimmune disease, curl pattern, hair disorder, hair texture, lupus erythematosus

## Introduction

Although ethnic variations in curl patterns have been well described, institution of curl pattern assessments in dermatologic examinations remain limited.^1^ Previous literature demonstrated alterations in curl patterns in African patients with autoimmune disorders, tuberculosis, and AIDS.^2^ However, the acquired hair textural changes associated with autoimmune hair disorders and the response to treatments remain poorly characterized. Herein, we report 3 cases with altered curl pattern following acute lupus alopecia (ALA) and alopecia areata (AA) with restoration of baseline hair texture after medical therapies.

## Case reports

### Case 1

**Fig 1:**
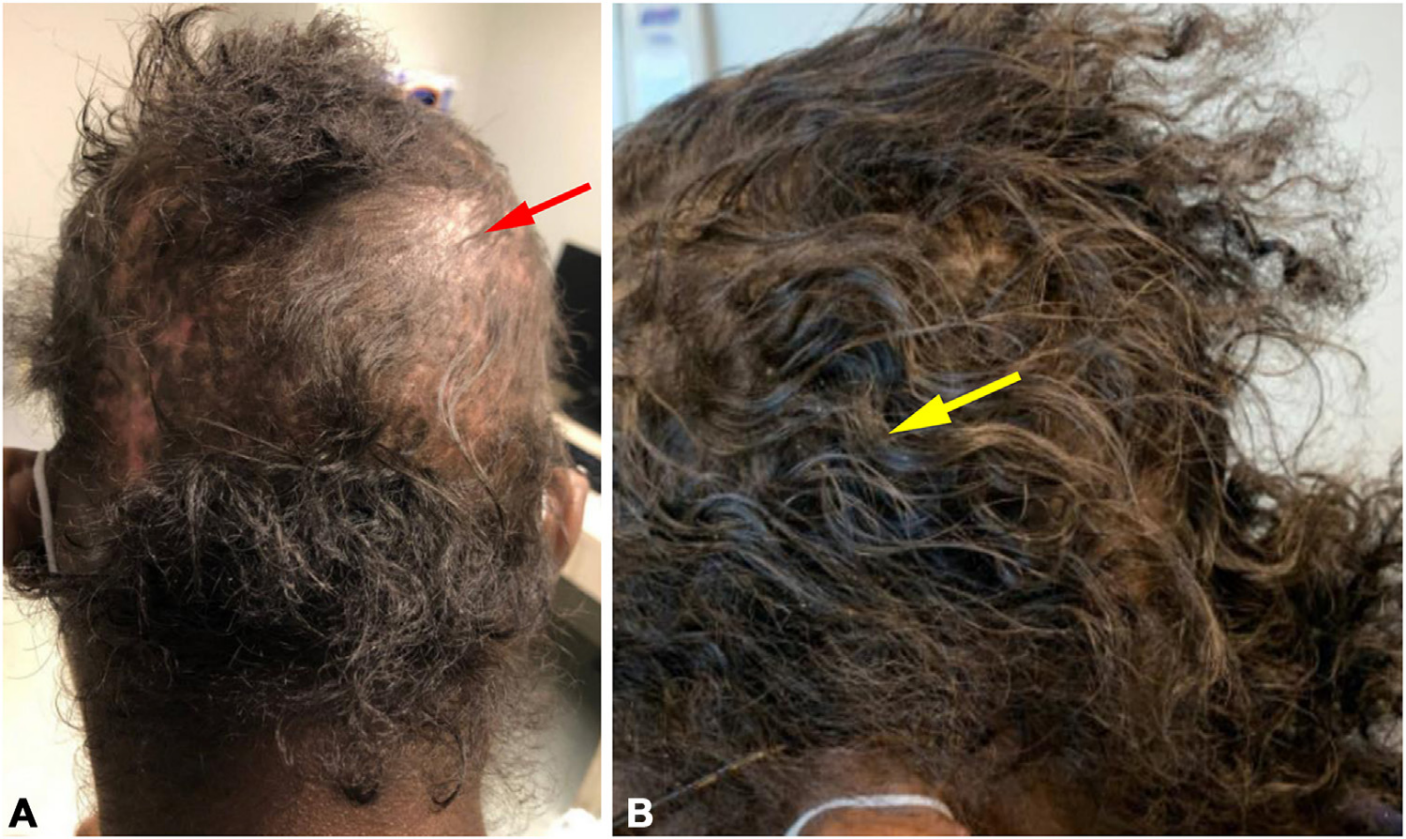
Acquired textural change in Afro-textured hair associated with acute lupus alopecia, with restoration of natural curl pattern after medical therapies. **A,** Pretreatment image of case 1 with acute lupus alopecia in the setting of systemic lupus erythematosus with lupus nephritis and discoid lupus erythematosus showing near complete loss of curl pattern and cotton-like appearance (*red arrow*). **B,** Posttreatment image of case 1 showing hair regrowth and restoration of natural curl pattern (*yellow arrow*) after 1 year of treatments with immunomodulators and intralesional and intramuscular corticosteroids.

A 23-year-old African American woman with an 11-year history of systemic lupus erythematosus (SLE) with concomitant discoid lupus erythematosus (DLE) and lupus nephritis was referred to our dermatology clinic for long-standing alopecia with recent hair loss episode after a SLE flare. Upon referral, the primary clinical concern was of areas with discoid lesions of the scalp, as the patient was noted to have retention of hair in other areas of the scalp. Her laboratory results were significant for elevated anti-dsDNA (≥1:640), anti-Smith, and anti-RNP antibodies and decreased complement levels. Scalp examination was notable for diffuse noncicatricial alopecia of the parieto-occipital scalp and discrete hypopigmented atrophic patches of cicatricial alopecia of the frontotemporal scalp, consistent with nonscarring diffuse alopecia secondary to SLE flare and DLE. Additionally, soft hair texture with near complete loss of curl pattern was noted and confirmed by the patient report (Fig 1, *A*). Following immunomodulatory therapies, including mycophenolate mofetil 3 g daily, hydroxychloroquine 400 mg daily, prednisone 25 mg daily, and voclosporin 23.7 mg twice a day along with intralesional and intramuscular triamcinolone acetonide, the patient demonstrated substantial hair growth with restoration of baseline curl pattern and thick texture after 1 year of treatment (Fig 1, *B*).

### Case 2

**Fig 2:**
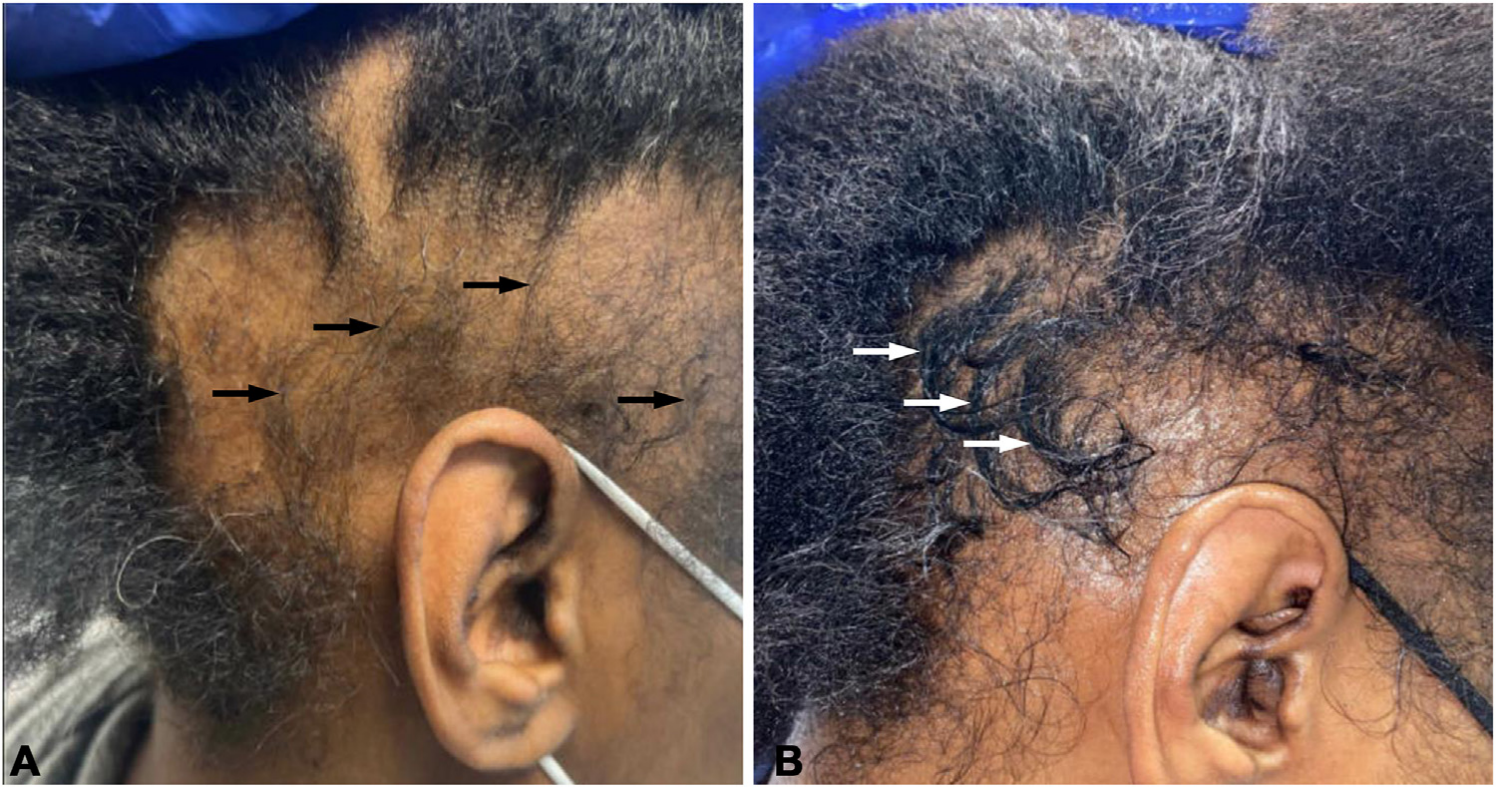
Restoration of curl pattern and texture in a patient with alopecia areata after treatments with corticosteroids. **A,** Pretreatment image of case 2 with patchy alopecia areata showing coalescing patches involving the right temporal and postauricular scalp with fine and loosely curled hair regrowth (*black arrows*). **B,** Posttreatment image of case 2 showing hair regrowth and partial restoration of curl pattern and texture (*white arrows*) after 6 months of treatments with topical, intralesional, and intramuscular corticosteroids.

A 41-year-old African American woman with a history of DLE presented for patchy hair loss on the scalp for 1 year. Scalp examination revealed well-demarcated nummular alopecic patches, save for thin downy hairs, involving the right temporal and postauricular scalp, consistent with AA (Fig 2, *A*). Following treatments with topical, intralesional, and intramuscular steroids, initial hair regrowth in the alopecic patches was noted to have a looser curl pattern than baseline (Fig 2, *B*). Continued treatment was associated with remission of AA and restoration of baseline texture and pattern over the course of 1 year.

### Case 3

**Fig 3:**
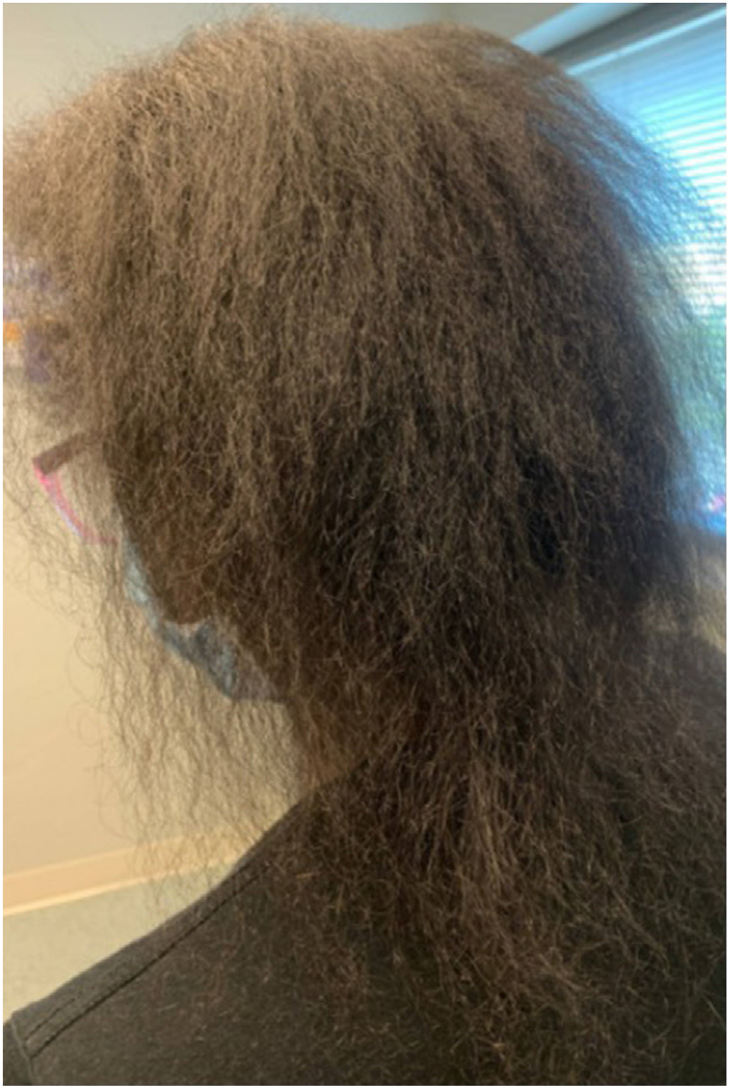
Acute lupus alopecia with curl pattern change in a patient without discoid lupus erythematosus or discrete alopecic patches. The altered appearance of hair and hair texture aided in the eventual diagnosis of systemic lupus erythematosus with associated proteinuria.

A 43-year-old African American woman with no known history of autoimmune disease presented with a complaint of active hair shedding. On examination, the patient was noted to have soft hair that was cotton-like in texture (Fig 3). No discrete alopecic or discoid patches or perifollicular inflammation were noted on examination. When probed, the patient admitted a change in hair curl pattern and texture as well as fatigue, joint pain, and recurrent oral ulcers. Her laboratory results were notable for leukopenia, high titers of antinuclear antibodies (>1:640) and anti-RNP antibodies (>643.8), low C4 level, proteinuria, and erythrocyte sedimentation rate of 56. Subsequently, a clinical diagnosis of SLE was established.

## Discussion

Autoimmune disorders can present with acquired textural alterations of afro-textured hair, especially in patients with ALA or AA. This finding joins other features, besides frank alopecia, that have been described in patients with autoimmune forms of hair loss. For example, poliosis has been documented as a clinical feature of AA and is associated with T-cell-mediated autoimmunity targeting the melanogenic zone of the hair bulb.^3^ In these patients, treatment until repigmentation of poliosis is recommended. Similarly, our cases suggest a potential clinical correlation between textural changes in African American hair and autoimmune disease activity, with restoration of hair texture following diminution of disease symptomatology. Regressive changes of decreased curvature in afro-textured hair may serve as a valuable sign for identifying susceptible patients to autoimmune disorders in this population. Compared with short vellus hair, characterized by clustered fine, nonpigmented hairs shorter than 10 mm commonly observed during the initial hair regrowth process, the “downy hair sign” may persist even after substantial improvement in hair density and length.^4^ Thus, monitoring for restoration of normal curl pattern and texture may be a helpful treatment end point.

In patients with ALA without discrete patches of DLE, a high index of clinical suspicion is required. As ALA often does not present with discrete patches of hair loss, curl pattern changes can be a helpful diagnostic clue. Given the historical prejudices and societal preferences for loose curl patterns, patients may not volunteer a change in their baseline curl pattern as this change may be considered preferable.^5^ The patient in case 3 highlights the degree of vigilance required for these patients as the clinical presentation can vary widely. Despite the abnormally soft, downy texture of her hair, without prompting from the clinician about changes in her curl pattern, autoimmune disease may not have been suspected. Unlike DLE, noncicatricial ALA is almost always associated with active SLE and specifically proteinuria >1 g/d.^6^ However, prognosis is favorable and treatment of systemic disease is key to improving outcomes. Similar clinical presentations may also be observed in severe presentations of rheumatoid arthritis and Behçet disease.^2^

Hair curliness is characterized by asymmetric differentiation of hair follicles programmed from the bulb^7^—which may be affected in AA and ALA. Hypotheses for curl pattern changes include inhibited activity of transglutaminase in the inner and outer root sheath, which is involved in the enzymatic process of cellular protein cross-linking, and α-keratin rearrangement of the inner root sheath during the disease process.^2^^,^^8^ Our case series is limited by the lack of data on the correlation between alteration in curl patterns and microscopic parameters, such as hair shaft diameter, cross-link density in the hair shaft, and light polarization. Further investigations using hair microscopy are warranted to delineate the morphologic features in autoimmune hair disorders. Future studies are required to elucidate the pathomechanism of hair textural changes associated with autoimmune disorders.

## Conflicts of interest

None disclosed.
